# The association between an early diagnosis of dementia and secondary health service use

**DOI:** 10.1093/ageing/afab079

**Published:** 2021-05-29

**Authors:** Elyse Couch, Christoph Mueller, Gayan Perera, Vanessa Lawrence, Matthew Prina

**Affiliations:** Institute of Psychiatry, Psychology, and Neuroscience, King’s College London, London, UK; Institute of Psychiatry, Psychology, and Neuroscience, King’s College London, London, UK; South London and Maudsley NHS Foundation Trust, London, UK; Institute of Psychiatry, Psychology, and Neuroscience, King’s College London, London, UK; Institute of Psychiatry, Psychology, and Neuroscience, King’s College London, London, UK; Institute of Psychiatry, Psychology, and Neuroscience, King’s College London, London, UK

**Keywords:** accident and emergency, dementia, early diagnosis, hospitalisation, mild cognitive impairment

## Abstract

**Background:**

dementia policy suggests diagnosing dementia early can reduce the risk of potentially harmful hospital admissions or emergency department (ED) attendances; however, there is little evidence to support this. A diagnosis of mild cognitive impairment (MCI) before dementia is a helpful proxy to explore early diagnosis. This study investigated the association between an early diagnosis of dementia and subsequent hospitalisations and ED attendances.

**Method:**

a retrospective cohort study of electronic health care records from 15,836 patients from a large secondary care database in South London, UK. Participants were divided into two groups: those with a diagnosis of MCI before dementia, an early diagnosis, and those without. Cox regression models were used to compare the risk of hospitalisation and ED attendance after dementia diagnosis and negative binomial regression models were used to compare the average length of stay and average number of ED attendances.

**Results:**

participants with an early diagnosis were more likely to attend ED after their diagnosis of dementia (HR = 1.09, CI = 1.00–1.18); however, there was no difference in the number of ED attendances (IRR = 1.04, CI = 0.95–1.13). There was no difference in the risk of hospitalisation (HR = 0.99, CI = 0.91–1.08) or length of stay between the groups (IRR = 0.97, CI = 0.85–1.12).

**Conclusion:**

the findings of this study do not support the assumption that an early diagnosis reduces the risk of hospitalisation or ED attendance. The patterns of health service use in this paper could reflect help-seeking behaviour before diagnosis or levels of co-morbidity.

## Key Points

- A previous diagnosis of mild cognitive impairment (MCI) is a useful proxy for an early diagnosis of dementia.
- An early diagnosis of dementia was associated with an increased risk of A&E attendance after diagnosis.
- There was no difference in the risk of hospitalisation between those with an early diagnosis and those without.
- There was no difference in the number of hospital days or A&E attendances between the groups.

## Introduction

The frequent use of emergency services and unplanned hospitalisations is reflective of fractured dementia care [1, 2]. It is not clear what steps need to be taken to reduce people living with dementia’s risk of hospitalisation or emergency department (ED) attendance. However, the early diagnosis of dementia has frequently been cited as a way of reducing the need for emergency care or hospitalisation [3]. All European countries with a national dementia strategy highlight the importance of receiving an early or ‘timely diagnosis’ of dementia, to enable people living with dementia to receive treatment and make advance care plans as early as possible to reduce the risk of unnecessary hospitalisations or ED attendances [4]. It is assumed that an early diagnosis of dementia can lead to a reduced risk of hospitalisation or use of emergency services; however, there is little empirical evidence to support this relationship [3, 5, 6].

There is no fixed definition for early diagnosis in dementia. Early diagnosis could be from the onset of neuropathology, many years before the symptoms become apparent, from the use of reliable predictive biomarkers or the onset of cognitive symptoms [5]. With the current state of evidence, it is possible to diagnose the pathologies that cause dementia early using predictive biomarkers; however, dementia is typically diagnosed in response to the onset of symptoms [5]. Mild cognitive impairment (MCI) is a diagnostic label commonly assigned to the early symptomatic stages of dementia where a full diagnosis cannot be confirmed [7]. Our previous research found people with a diagnosis of MCI before dementia have less severe cognitive, psychiatric and functional symptoms at dementia diagnosis. This profile of symptoms is consistent with the early stages of dementia; therefore, a previous diagnosis of MCI is a useful proxy for the early diagnosis of dementia [8].

### Aims

In theory, people with an early diagnosis should receive early treatment, have more contact with primary health services ahead of time and be supported to make advanced plans, which reduce the risk of hospitalisation or ED attendance [6]. However, it is unclear whether this happens. Therefore, the primary aim of this study was to examine whether there is any difference in the risk of hospitalisation or ED attendance between participants with an early diagnosis, as defined by a previous diagnosis of MCI, and those without. Secondly, we examined whether the length of stay and number of ED attendances differed between the two groups.

## Methods

To address the aims of this study, we conducted a retrospective cohort study using electronic health records from South London and Maudsley NHS Foundation Trust (SLaM). SLaM provides specialist dementia care to people living with dementia in the London boroughs of Lambeth, Lewisham, Southwark and Croydon.

### Data sources and linkages

Data from SLaM's electronic medical health care records were extracted through SLaM's Biomedical Research Centre Clinical Record Interactive Search (CRIS). Data are stored in both free text and structured fields, the extraction of which has been previously described [9, 10]. Additionally, we used an existing linkage between CRIS and NHS Digital Health Episode Statistics (HES) to extract data on hospitalisations and visits to ED. HES data were available until 31 March 2017.

The CRIS database has full approval for secondary analysis (Oxford Research Ethics Committee C, reference: 08/H0606/71 + 5).

### Participants

Participants were included in the cohort if they received a diagnosis of dementia according to ICD 10 classifications [11], between 2 January 2008 and 30 March 2016, and were over the age of 50. The first diagnosis of dementia served as the index date and all participants had at least 1 year of HES follow-up data available.

### Measures

Participants with a diagnosis of MCI, as recorded by an ICD-10 code of F06.7, before the index date were classified as having received an ‘early diagnosis’. This was included as a dichotomous variable.

Our primary outcomes of interest were time to first hospitalisation and time to first ED attendance. Our secondary outcomes of interest were the cumulative number of hospital days and number of ED attendances.

As co-variates, we extracted whether participants were hospitalised or attended ED in the year before dementia diagnosis, as these are known predictors of ED attendance/hospital admission after diagnosis [12]. Demographic information from the time of dementia diagnosis were extracted including age, gender, ethnicity (coded as European, Black, Asian or Other), marital status and levels of social deprivation. A raw score for neighbourhood index of social deprivation was estimated using the participant’s most recent address [13]. Participant’s Mini-Mental State Exam (MMSE) scores, which rates the severity of cognitive impairment on a scale of 1–30 (where a higher score indicates less cognitive impairment) [14], at the time of dementia diagnosis were extracted. Participant’s scores on the HoNOS 65+, which rates functional and other psychiatric symptoms, were extracted at the time of diagnosis. The number of psychiatric symptoms experienced by participants was grouped by number of symptoms: no symptoms, 1 symptom, 2 symptoms and 3 or more symptoms. We also extracted whether participants were prescribed AChIEs within 6 months of diagnosis, and this was dichotomised.

### Statistical analysis

All analyses were conducted using Stata 15 [15]. *T*-tests and Chi-squared test were used to compare baseline differences between the early diagnosis and no early diagnosis groups.

We assessed the risk of hospitalisation and ED attendance after dementia diagnosis using cox regression models. Negative binomial regression models were used to compare the length of stay (number of days) and the number of ED attendances by each group. We used negative binomial regression, rather than Poisson regression, as data were over-dispersed. We present an unadjusted model and a multivariable model adjusted for age, gender, ethnicity, physical illness, marital status, prescription of ACHEIs, number of psychiatric symptoms, MMSE scores and previous hospitalisation/ED attendance. Follow-up time was included in both models as an exposure variable.

### Missing data

Thirty percent of participants were missing MMSE scores and 13% of participants were missing one or more scores on the HoNOS 65+. Missing data were imputed in STATA using multiple imputation by chained equations [16]. All outcomes and co-variates were included in the imputation.

## Results

### Demographics

We identified 15,836 people with dementia; 5.1% of participants (*n* = 807) were diagnosed with MCI before they were diagnosed with dementia. Table 1 presents the characteristics of included participants. Participants with an early diagnosis were more likely to be white, to be prescribed ACHEIS, have higher levels of social deprivation, and have impaired cognition and activities of daily living. A greater proportion of participants with an early diagnosis attended ED before their diagnosis of dementia than those without.

**Table 1:** Characteristics of included participants

Demographic information at dementia diagnosis	All participants (*N* = 15,836)	Early diagnosis (*N* = 807)	No early diagnosis *N* = (15,029)	*P*
Gender (%)				0.99
Male	39.18	39.16	39.18	
Female	60.82	60.82	60.82	
Ethnicity (%)				>0.01^*^
European (British, Irish, etc.)	74.67	79.45	74.41	
Black (Caribbean, African, other)	16.49	14.82	16.58	
Asian (Indian Bangladesh, other Asian)	4.51	2.99	4.59	
Other	4.33	2.74	4.42	
MCI diagnosed before dementia (%)	5.10			
Mean Age (SD)	80.84 (8.64)	80.64 (8.19)	80.85 (8.67)	0.49
Mean MMSE score (SD)	18.52 (6.30)	21.51 (5.74)	18.36 (6.29)	>0.01^*^
Mean index of deprivation (SD)	27.30 (11. 06)	28.60 (10.20)	27.24 (11.11)	>0.01^*^
Prescribed AChEIs 6 months ± dementia diagnosis (%)	32.49	39.78	32.10	>0.01^*^
Marital status (%)				0.66
Current partner	33.68	32.95	33.72	
No current partner	66.32	67.05	66.28	
HoNOS65+ psychiatric symptoms (%)				0.13
No symptoms	35.06	38.79	34.86	
1 symptom	29.94	29.24	29.98	
2 symptoms	18.46	16.85	18.54	
3+ symptoms	16.54	15.12	16.62	
HONOS65+ activities of daily living (%)	62.13	55.67	62.47	>0.01^*^
HoNOS65+ physical illness and disability (%)	56.17	55.07	56.23	0.55
Health service use in year before dementia diagnosis				
Attended ED (%)	70.34	73.94	70.15	0.03^*^
Was hospitalised (%)	54.79	54.40	54.81	0.82

^*^ *P* < 0.05

### Risk of hospitalisation or ED attendance

Most participants had a hospitalisation (74%) recorded after they were diagnosed with dementia (Table 2). The median time to first hospitalisation after dementia diagnosis was 11.5 months. Adjusted and unadjusted cox regression models showed there was no significant difference in the risk of hospitalisation between the groups.

**Table 2:** Cox regression models comparing time to first hospitalisation and ED attendance after dementia diagnosis between early diagnosis and no early diagnosis group

Outcome	%	Median time to outcome (year)	Risk of outcome			
			Unadjusted HR (95% CI)	*P*	Adjusted HR^a^ (95% CI)	*P*
Hospitalisation						
All participants	73.96	0.91 (0.27–2.47)				
Early diagnosis	71.50	0.87 (0.28–2.63)	0.96 (0.88–1.04)	0.35	0.99 (0.91–1.08)	0.76
No early diagnosis	74.09	0.91 (0.27–2.46)	Ref		Ref	
ED attendance						
All participants	75.73	0.85 (0.26–2.28)				
Early diagnosis	75.22	0.73 (0.23–2.29)	1.03 (0.95–1.11)	0.53	1.09 (1.00–1.18)	0.04^*^
No early diagnosis	75.75	0.85 (0.26–2.28)	Ref		Ref	

^a^Models adjusted for: age, gender, ethnicity, marital status, MMSE scores at dementia diagnosis, co-morbid physical conditions, prescription of ACHEIs, activities of daily living, psychiatric symptoms and hospitalisation/ED attendance before dementia diagnosis

^*^ *P* < 0.05. Note: No early diagnosis used as reference group.

Over two-thirds of participants attended ED after their dementia diagnosis (75.7%). The median time to first ED attendance in the early diagnosis group was 8.9 months, compared with 10.6 months. Adjusted cox regression models showed participants with an early diagnosis were at increased risk of attending ED (HR = 1.09, CI = 1.00–1.18, *P* = 0.4).

### Length of stay and number of ED attendances

Table 3 presents the mean number of hospital days and ED attendances per 100 person years. Participants with an early diagnosis had a significantly shorter length of stay at 10.8 hospital days compared with 10.27 hospital days (*P* = 0.01). There was no significant difference in number of ED attendances between the groups.

**Table 3:** Mean number of ED attendances and hospital days per 100 person years and negative binomial regressions comparing length of stay and number of ED attendances between early diagnosis and no early diagnosis group

Outcome	Mean number per 100 person years (95% CIs)	IRR (95% CI)			
		Unadjusted	*P*	Adjusted^a^	*P*
Hospital days					
All participants	10.26 (10.24–10.29)				
Early diagnosis	10.08 (9.95–10.21)^*^	0.89 (0.76–1.01)	0.08	0.97 (0.85–1.12)	0.70
No early diagnosis	10.27 (10.24–10.31)	Ref		Ref	
ED attendances					
All participants	1.22 (1.21–1.23)				
Early diagnosis	1.26 (1.20–1.31)	1.02 (0.93–1.11)	0.68	1.04 (0.95–1.13)	0.38
No early diagnosis	1.22 (1.21–1.23)	Ref		Ref	

^a^Models adjusted for: age, gender, ethnicity, marital status, MMSE scores at dementia diagnosis, co-morbid physical conditions, prescription of ACHEIs, activities of daily living and psychiatric symptoms and follow-up time

^*^ *P* < 0.05. Note: No early diagnosis used as reference group.

Negative binomial regressions, adjusted for a range of confounders, showed there was no difference in the count of hospital days between the groups (IRR = 0.97, CI = 0.85–1.12). Similarly, there was no difference in the count of ED attendances (IRR = 1.04, CI = 0.95–1.13).

## Discussion

In this study, we investigated whether an early diagnosis was associated with a decreased risk of hospitalisation or ED attendance after a diagnosis of dementia. We found that participants with an early diagnosis were at greater risk of attending ED than participants without an early diagnosis; however, there was no difference in the number of ED attendances between the groups. There was no difference in the risk of hospitalisation or length of stay between participants with an early diagnosis and those without.

We found a high level of secondary health service use in people with dementia, 74% of participants were hospitalised and 75% attended ED after their diagnosis. The average time to the first hospitalisation and first ED visit was 11.5 and 10.4 months, respectively. This is consistent with previous research, which showed that people living with dementia have high rates of admission to hospital within the first year of diagnosis [1]. These are important findings, as the early or timely diagnosis of dementia is a cornerstone of dementia policy in the UK and Europe [4]. Our findings suggest that an early diagnosis, or early help-seeking, alone is not sufficient to reduce the need for potentially harmful hospitalisations and ED attendances. This indicates that we need to think beyond diagnosing dementia early. We do not currently understand how to reduce hospitalisation and ED attendance in people living with dementia. Future research should investigate how post-diagnostic support from health and community services can reduce the risk of using secondary health care services.

We found, contrary to popular belief, that the risk of hospitalisation and length of stay did not differ between people with an early diagnosis of dementia compared to those without. Additionally, people with an early diagnosis had a higher risk of attending ED, although there was no difference in the number of times each group attended ED. This group may have had increased contact with health services before their diagnosis of dementia, which increased the likelihood of receiving the early diagnosis of dementia, and this pattern of health service use continued after diagnosis.

Many hospital admissions for people living with dementia are necessary and appropriate. However, people living with dementia are at greater risk of negative outcomes arising from hospitalisation than older adults of the same age without dementia. They may be hospitalised for longer [17, 18], may be less likely to be given appropriate treatment or pain relief [18–20], can experience significant cognitive decline during their admissions [21] and are at greater risk of developing delirium [18, 22]. Similarly, people living with dementia use ED more than older adults of the same age [23]. ED visits can be difficult for people living with dementia and their carers; they require additional care for their illness and extra support to cope with the unfamiliar environment in ED. ED visits for people living with dementia are also likely to increase in the last few months of life and are more likely to be emergency referrals, by ambulance or out of hours, indicating visits are made at a time of crisis [2]. It is important that people living with dementia are able to access the health services they need at the time they need it; however, more research is needed to understand how to reduce the risk of unnecessary hospitalisation and ED use by people living with dementia.

There is a risk that focusing on diagnosing dementia early and investing in treatments for the early stages of the disease diverts resources from meeting other needs in the later stages, including the treatment of co-morbidities [6]. Previous research has found that people living with dementia tend to access services for their co-morbid conditions, rather than for their dementia [1], and an increased number of co-morbid conditions is associated with increased primary and secondary health service use [24]. Over half of the participants included in this study had high levels of co-morbid physical illness or disability. It is possible that there is no difference in risk of hospitalisations between the two groups because they have similar levels of co-morbid conditions and are therefore accessing services in a similar way. It is not clear how a diagnosis of dementia affects the treatment of co-morbid conditions; however, there is evidence that services should take a more holistic approach to treating dementia and co-morbid conditions in the hope of reducing hospital admissions and ED visits [24, 25].

### Limitations

The cohort from this study came from a secondary care database, which reflects the high levels of service use. Further research is needed to understand the impact of an early diagnosis or early help-seeking on the use of other types of health services, such as primary care. While we have highlighted the possible role of co-morbidities in driving high levels of health service use, our data are restricted to HoNOS rated levels of co-morbidities without information on individual conditions. This is an interesting avenue for future research. This is a cohort study; therefore, variables used in this study were limited to what is routinely collected, and there may be some residual confounding, which has not been controlled for. While we have previously found a previous diagnosis of MCI to be a useful proxy for early diagnosis [8], we cannot be conclusive that participants in the early diagnosis group were diagnosed earlier in the disease. Furthermore, in this study, we were not able to differentiate between necessary and avoidable hospitalisations or ED attendances. Finally, the negative findings make it difficult to draw conclusions for clinical practice; however, they do have implications for policies that promote the benefits of diagnosing dementia early.

### Implications and directions for future research

We have found that early diagnosis alone is not a preventative step for reducing hospitalisations or ED attendances and people with an early diagnosis had an increased risk of attending ED. However, an equal or higher use of health services between people with an early diagnosis and those without is not necessarily a bad thing. People living with dementia should be able to access appropriate health services whenever they are needed. However, people with dementia are at greater risk of negative outcomes following a hospitalisation or ED attendances [18, 23] and should probably be avoided in lieu of other types of support. Previous research in the United States has shown that people living with dementia tend to use medical services, rather than other community care services [26]. Future research is needed to understand the differences in health service and community social care use between people who are diagnosed with dementia, taking co-morbid health conditions, the availability of post-diagnostic services and previous patterns of health service use into consideration. It is important to understand where services are being under- or over-utilised—and why—to make them more responsive to the needs of people living with dementia.
